# Optimization of Extraction of Circulating RNAs from Plasma – Enabling Small RNA Sequencing

**DOI:** 10.1371/journal.pone.0107259

**Published:** 2014-09-17

**Authors:** Melanie Spornraft, Benedikt Kirchner, Bettina Haase, Vladimir Benes, Michael W. Pfaffl, Irmgard Riedmaier

**Affiliations:** 1 Physiology Weihenstephan and ZIEL Research Center for Nutrition and Food Sciences, Technical University Munich, Freising, Germany; 2 Genomics Core Facility, EMBL European Molecular Biology Laboratory, Heidelberg, Germany; CNRS UMR7622 & University Paris 6 Pierre-et-Marie-Curie, France

## Abstract

There are several protocols and kits for the extraction of circulating RNAs from plasma with a following quantification of specific genes via RT-qPCR. Due to the marginal amount of cell-free RNA in plasma samples, the total RNA yield is insufficient to perform Next-Generation Sequencing (NGS), the state-of-the-art technology in massive parallel sequencing that enables a comprehensive characterization of the whole transcriptome. Screening the transcriptome for biomarker signatures accelerates progress in biomarker profiling for molecular diagnostics, early disease detection or food safety. Therefore, the aim was to optimize a method that enables the extraction of sufficient amounts of total RNA from bovine plasma to generate good-quality small RNA Sequencing (small RNA-Seq) data. An increased volume of plasma (9 ml) was processed using the Qiagen miRNeasy Serum/Plasma Kit in combination with the QIAvac24 Plus system, a vacuum manifold that enables handling of high volumes during RNA isolation. 35 ng of total RNA were passed on to cDNA library preparation followed by small RNA high-throughput sequencing analysis on the Illumina HiSeq2000 platform. Raw sequencing reads were processed by a data analysis pipeline using different free software solutions. Seq-data was trimmed, quality checked, gradually selected for miRNAs/piRNAs and aligned to small RNA reference annotation indexes. Mapping to human reference indexes resulted in 4.8±2.8% of mature miRNAs and 1.4±0.8% of piRNAs and of 5.0±2.9% of mature miRNAs for *bos taurus*.

## Introduction

Since the discovery of microRNAs (miRNAs) in *c.elegans* in the year 1993, the research in the world of small non-coding RNAs (ncRNAs) increased drastically. Due to the finding of novel RNA classes that have no protein-coding potential and do not overlap with already characterized non-coding RNA species, there are new transcript populations with various sizes and functions. Their role in RNA modification and silencing needs to be further unraveled. NcRNAs which are important for protein translation like tRNA and rRNA or RNAs responsible for RNA function and maturation e.g. small nuclear RNAs (snRNA) or small nucleolar RNAs (snoRNA) are well known and intensively investigated for decades. In the last years, additional species of ncRNAs have been discovered and their examination is very popular in recent science. MiRNAs and PIWI-interacting RNAs (piRNAs) as members of the RNA interference (RNAi) group play a major role in this context.

MiRNAs have a length of 19–25 nucleotides (nts) and negatively regulate gene expression by translational inhibition of mRNAs or by mRNA degradation. In the year 2000, miRNAs were detected in humans and over the years, correlations between miRNAs and diseases like cancer and cardio-vascular diseases were discovered [1]. In 2008, miRNAs were found in plasma and one year later they were used as plasma markers for cardiac disease in clinical diagnostics [1].

PiRNAs are longer than miRNAs (25–32 nts) and bind to the piwi- (P-element induced wimpy testes)/argonaute protein family, whose functions were primarily discovered in a *Drosophila*-mutant [2]. The PIWI-piRNA-complex is mandatory in gametogenesis as it is linked to germline and stem cell development as well as to gene silencing by regulating transposons [3]. However, there are few publications about the appearing of piRNAs in organisms, the piRNA distribution in different organs and fundamental research needs to decode their biological function.

Profiling of serum or plasma and/or other body fluids (e.g. urine, breast milk, saliva) for regulating small RNAs can provide new biomarkers for a broad range of diseases and biological processes. Their analysis offers various advantages which could make them a goldmine in the identification of novel biomarkers:

- Small RNAs are found in non- or minimally invasive specimens simplifying sampling for both the clinician and the patient compared to tissue sampling via biopsies.
- They are relatively stable in clinical samples regarding RNase digestion, temperature variation and multiple freeze-thaw cycles [4], [5].
- The detection of small RNA biomarkers is already proven in different illnesses, e.g. in cancer [5], [6], cardiovascular diseases [7], myocardial infarction [8] and central nervous system diseases [9].
- Nowadays, analysis of ncRNAs is a highly updated research field. The interest in the survey of their post-translational actions is massive as they show great promise for new insights in cell biology [10].

In the search for circulating nucleic acids plasma biomarkers, mainly miRNAs are in the focus of investigation. The commonly used strategy therefore is using microarrays but mainly RT-qPCR [11], [12]. However, the number of analyzed miRNAs is limited when working with these methods and the RNAs of interest need to be known before the experiment. NGS offers researchers a completely detailed view into their samples. All transcripts are captured in an integral picture of the sample. In terms of throughput, sequence output, data generation rate and data quality, sequencing performance is continuously increasing while costs, hands-on time in the lab and sequencing durations are decreasing. That's why NGS is considered to be the state-of-the-art technology when it comes to holistic gene expression profiling. However, detecting circulating nucleic acids biomarker profiles in plasma by NGS and especially by small RNA-Seq is in its infancy.

The amount of RNA which can be extracted with purchasable kit systems for circulating RNAs from body fluids is only sufficient for a limited number of RT-qPCR analyses, but not for the application of small RNA-Seq. Therefore, small RNA-Seq biomarker profiling in body fluids is very challenging. Commercially available kits for RNA isolation from bio-fluids use low starting volumes: 50–200 µl (miRNeasy Serum/Plasma Kit, Qiagen, Germany) or 300 µl (NucleoSpin miRNA Plasma Kit, Macherey-Nagel, Germany). Standard methods for measuring RNA yield with UV-Vis spectrophotometric instruments like Nano Drop (Thermo Scientific, Germany) are not optimally suited for measurements below the detection limit of 2 ng/µl of RNA. Moreover, using the Small RNA Analysis Kit on the Bioanalyzer 2100 (Agilent Technologies, Germany) to resolve and quantify the small nucleic acid fraction of extracted RNAs does also not provide signals in electropherograms. Furthermore, reverse transcription reactions request a consistent input amount of extracted RNA, which is difficult as long as the concentration cannot be reliably measured. In addition, contamination of plasma samples with cellular RNA from lysed cells (e.g. apoptotic epithelial cells in urine or ruptured red blood cells during sampling) can alter the RNA expression profile and disturb robust biomarker detection [13].

Besides profiling of clinical samples for disease-specific biomarkers, the screening for distinct and convincing RNA signatures is an applied method in the surveillance of food safety and anti-doping control systems in food-delivering livestock as well as in human or equine competitive sports [5], [14], [15]. Including small RNAs in the screening for differentially expressed genes on the transcriptional level could lead to multiple RNA biomarkers that are more robust, reliable and failure-safe. Here, an adaption of an RNA isolation method is presented that enabled the holistic analysis of circulating cell-free miRNAs and piRNAs in bovine plasma via small RNA-Seq.

## Materials and Methods

### Plasma sampling

Blood was taken from *vena jugularis* from nine male Holstein Friesian calves at the age of 6 months. For blood drawing, 9 ml K3E K_3_EDTA-Vacuette tubes (Greiner bio-one, Germany) and single use needles (20G x 1½”, Greiner bio-one, Germany) were used. Plasma was separated from other blood components directly after blood sampling (15 min for 3500×rcf at room temperature with transportable centrifuges EBA20, Hettich, Germany) and stored at -80°C until RNA extraction. Blood collection was approved by the ethical committee of the Landesamt für Natur, Umwelt und Verbraucherschutz Nordrhein-Westfalen (Recklinghausen, Germany) (permit number 84-02.04.2012.A040). Animals were housed and fed according to good animal attendance practice and all efforts were made to minimize suffering.

### Total RNA extraction

Frozen plasma samples were used for small RNA extraction. It is important to consider the existence of contaminations containing RNAs such as intact cells, apoptotic cells or cell fragments. The presence of cells or lysis of blood cells with a following release of their content to plasma can alter the RNA expression profile. Thus, after thawing, plasma samples were centrifuged at 3000×rcf for 5 min at room temperature to pellet debris.

To extract circulating RNA species from plasma samples, the miRNeasy Serum/Plasma Kit (Qiagen, Germany) was used with modifications. Instead of 200 µl of initial sample material, a volume of 9 ml was applied. To improve the handling of increased volumes and to prevent multiple column loadings, the resulting column clogging, the need for bigger consumables, e.g. centrifugation tubes, and more needed time, the vacuum device QIAvac24 Plus (Qiagen, Germany) was used. 9 ml were separated in 3×3 ml fractions and transferred to 10 ml plastic centrifuge tubes with push caps (Sarstedt, Germany). The amount of cell lysis reagent QIAzol (Qiagen, Germany) and chloroform (AppliChem, Germany) was raised accordingly to 9 ml and 6 ml, respectively. 3 ml of QIAzol were added to each centrifuge tube, mixed by vortexing and incubated at room temperature for 5 min. 2 ml chloroform were added to each tube, vortexed for 15 s and incubated at room temperature for 3 min. An EBA20 benchtop centrifuge (Hettich, Germany) was used in a 4°C cooling chamber for the phase separation steps (15 min, 3500×rcf). After centrifugation, the mixture separates clearly into three phases: the upper aqueous, transparent phase containing RNA, the white interphase and the lower organic, reddish phase with proteins. The aqueous phases of the three separated samples were pooled in a 50 ml reaction tube followed by addition of 1.5 volumes of ethanol (100%) and transfer to one spin column. A vacuum pump was connected to the QIAvac24 Plus vacuum manifold equipped with luer plugs, RNeasy MinElute spin columns and tube extenders (Qiagen, Germany). Washing and binding buffers were applied twice. RNA was eluted with an increased volume of RNase-free water (50 µl) by applying two times 25 µl. To compare RNA yields from the presented method with those from purchasable kits, RNA was extracted from plasma samples using the miRNeasy Serum/Plasma Kit (Qiagen, Germany), the NucleoSpin miRNA Plasma Kit (Macherey Nagel, Germany) and the miRNeasy Mini Kit (Qiagen, Germany) with their recommended supplementary miRNA extraction protocol. If an initial plasma volume other than stated in the manufacturer's specifications was tested, multiple column loadings were necessary to process samples.

### RNA Quantity and Quality Check

Subsequently, RNA yields were measured using the Qubit 2.0 Fluorometer (Life Technologies, Germany) in combination with the RNA Assay Kit (Life Technologies, Germany) according to the manufacturer's protocol. The maximum volume of sample input was used (20 µl), standards were freshly prepared and the Qubit was equilibrated after the manufacturer's instructions. A Bioanalyzer 2100 (Agilent Technologies, Germany) run using the Small RNA Kit (Agilent Technologies, Germany) was performed afterwards for the analysis and quantification of RNA eluates resolving small RNAs in the range from 6 to 150 nts length. The extracted RNA was stored at −80°C until further analysis.

### Library Preparation and small RNA Sequencing

For sequencing, the small RNA transcripts were converted into barcoded cDNA libraries. Library preparation was performed with the NEBNext Multiplex Small RNA Library Prep Set for Illumina (New England BioLabs Inc., USA) followed by small RNA-Seq analysis on the Illumina HiSeq2000 platform (Illumina Inc., USA). Limited RNA quantity led to library preparation with 35 ng of RNA as starting material. Multiplex adaptor ligations, reverse transcription primer hybridization, reverse transcription reaction and the PCR amplification were processed with regard to the protocol for library preparation (Protocol E7330, New England BioLabs Inc., USA). When working with lower RNA input, the protocol offers modifications at several steps, for example a longer incubation time and reduced temperatures in the adaptor ligation step. These modifications are increasing the ligation efficiency of methylated RNAs such as piRNAs. Therefore, the reaction was carried out for 18 h at 16°C instead of 1 h at 25°C. After PCR pre-amplification, the cDNA constructs were purified with the MinElute PCR Purification Kit (Qiagen, Germany) and loaded on the Bioanalyzer 2100 (Agilent, Germany) using the DNA 1000 Kit (Agilent, Germany) according to the manufacturer's protocol. For the size selection of amplified cDNA libraries, PCR products were loaded on an agarose gel (4%), appropriate bands of approximately 135 bp to 160 bp in size were cut out and passed on to gel extraction with the MinElute Gel Extraction Kit (Qiagen, Germany). A concluding Bioanalyzer 2100 run with the High Sensitivity DNA Kit (Agilent Technologies, Germany) that allows the analysis of DNA libraries regarding size, purity and concentration completed the workflow of library preparation. The obtained sequence libraries were subjected to the Illumina sequencing pipeline, passing through clonal cluster generation on a single-read flow cell (Illumina Inc., USA) by bridge amplification on the cBot (TruSeq SR Cluster Kit v3-cBOT-HS, Illumina Inc., USA) and 50 cycles sequencing-by-synthesis on the HiSeq2000 (Illumina Inc., USA).

### Software, Statistics and Mapping

To assess overall NGS data quality, adaptor sequences were trimmed from the 3′end using Btrim [16] and reads without detectable adaptors were excluded from the data set. Sequence length distribution as well as the base calling accuracy as indicated by the phred quality score (Q score) were calculated with high throughput sequence data quality control software FastQC (Babraham Bioinformatics, UK, Version 0.10.1). To avoid distortion and generation of false positive mappings by degraded RNA material and other small reads, the data set was depleted of all reads with a sequencing length of less than 16 nts for miRNA and 26 nts for piRNA analysis respectively. In addition, all rRNA, tRNA, snRNA as well as snoRNA reads were omitted using their corresponding bovine sequences obtained from Rfam database [17] prior to mapping to further improve specificity of the read data.

All mappings including the previous Rfam comparison were done via Bowtie short read aligner [18] using the default parameters with the exception of the “best” alignment algorithm and only allowing one mismatch in the first 15 nts. Aligned reads were then sorted and indexed by SAMtools [19] and final readcounts were generated by calling the sum of hits per miRNA or piRNA sequence respectively. To obtain miRNA readcounts, the trimmed and filtered reads were aligned to the most recent miRBase database of mature miRNAs ([20], release 20) for *bos taurus* (bta) and *homo sapiens* (hsa). Likewise for piRNA readcounts, reads were first subjected to an additional filtering on the complete miRBase database of mature miRNAs. To generate a bowtie index for piRNA alignment, the nucleotide database of NCBI Genbank ([21], Release 201) was searched for human piRNA entries (32.046 entries in February 2014) that were merged to a file of short piRNA sequences. In addition, reads mapped on this index were checked for piRNA specific traits. A sequence motif analysis was done to evaluate 5′-T-bias by creating a positional weight matrix of all mapped piRNA reads using R and the packages ShortRead, Biostrings and seqLogo (http://www.bioconductor.org/). Furthermore, reads were mapped on the bovine genome (NCBI UMD 3.1, [22]) using bowtie and tested for ping-pong-cycle formation using a python script developed by Antoniewski [23]. To detect piRNA clusters in the bovine genome, piRNA reads were mapped with SeqMap [24] allowing no mismatches and resulting Eland3-format data from all nine animals was combined (NGS tools for the novice, http://www.uni-mainz.de/FB/Biologie/Anthropologie/487_ENG_HTML.php). ProTRAC software was used with the default settings but random base composition was chosen and the minimal score for accumulation of loci with typical length was set to zero to analyze and visualize clusters [25].

Although bovine plasma was used, the analysis of reads that were mapped to the human reference databases is necessary. First, hsa mapping results are crucial for the evaluation of piRNAs as there are -up to now- no annotated piRNAs for bovines. Second, the mapping output for hsa consisted of 2.578 entries for mature miRNAs and of 32.046 entries for piRNAs, whereas the bta output consisted of 783 entries for mature miRNAs and of none for the bovine piRNAs. Sequenced reads were deposited in the European Nucleotide Archive ENA (http://www.ebi.ac.uk/ena/, accession numbers PRJEB6683/ERP006244).

## Results

### RNA Quantity and Quality

RNA yield of plasma samples that were extracted with a choice of commercially available kits were not quantifiable due to a too low amount of RNA (Table 1). Although the amount of sample input was increased in the tested kits during the assay optimization process, RNA was still not measureable. Only by using the miRNeasy Serum/Plasma Kit (Qiagen, Germany), experiments with a raised plasma input (6 ml) showed quantifiable RNA concentrations (Table 1). After optimization, the miRNeasy Serum/Plasma Kit coupled with the QIAvac System was applied to isolate total RNA from 9 ml of nine different bovine plasma samples. Yields were 77.4 ng±24.9 ng which were determined by fluorometric quantification (Table 1).

**Table 1:** Optimization process of total RNA extraction.

Isolation system	Plasma input [ml]	RNA yield [ng]	Sample
Assay optimization			
NucleoSpin miRNA Plasma Kit (Macherey-Nagel, Germany)	0.3	not quantifiable	
NucleoSpin miRNA Plasma Kit (Macherey-Nagel, Germany)	0.6	not quantifiable	
NucleoSpin miRNA Plasma Kit (Macherey-Nagel, Germany)	1.0	not quantifiable	
miRNeasy Kit, supplementary control (Qiagen, Germany)	0.3	not quantifiable	
miRNeasy Kit, supplementary control (Qiagen, Germany)	0.6	not quantifiable	
miRNeasy Kit, supplementary control (Qiagen, Germany)	1.0	not quantifiable	
miRNeasy Serum/Plasma Kit (Qiagen, Germany)	6.0	37.2	
miRNeasy Serum/Plasma Kit (Qiagen, Germany)	6.0	61.0	
**Analyzed samples**			
miRNeasy Serum/Plasma Kit + QIAvac System (Qiagen, Germany)	9.0	57.6	Animal 1
miRNeasy Serum/Plasma Kit + QIAvac System (Qiagen, Germany)	9.0	36.3	Animal 2
miRNeasy Serum/Plasma Kit + QIAvac System (Qiagen, Germany)	9.0	72.9	Animal 3
miRNeasy Serum/Plasma Kit + QIAvac System (Qiagen, Germany)	9.0	38.4	Animal 4
miRNeasy Serum/Plasma Kit + QIAvac System (Qiagen, Germany)	9.0	45.0	Animal 5
miRNeasy Serum/Plasma Kit + QIAvac System (Qiagen, Germany)	9.0	51.9	Animal 6
miRNeasy Serum/Plasma Kit + QIAvac System (Qiagen, Germany)	9.0	112.0	Animal 7
miRNeasy Serum/Plasma Kit + QIAvac System (Qiagen, Germany)	9.0	71.9	Animal 8
miRNeasy Serum/Plasma Kit + QIAvac System (Qiagen, Germany)	9.0	210.4	Animal 9

The table compiles the tested plasma input volumes, the used isolation systems and the resulting yields of extracted total RNA [ng] measured with the Qubit 2.0 Fluorometer.

**Figure 1:**
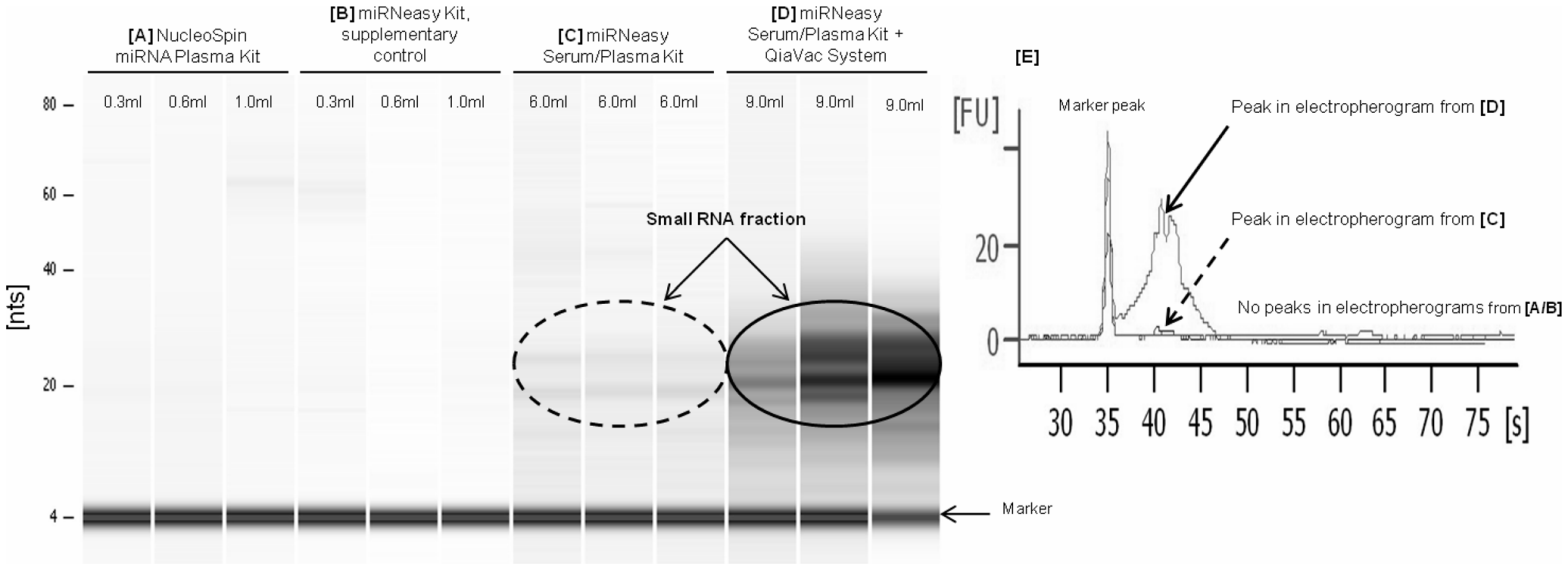
Small RNA Series II Bioanalyzer assay as checkpoint for RNA quantity after extraction procedure. Intensity of bands in the gel images ([A–D]) depicts the quantity of eluted RNAs with different isolation systems and varying plasma input volumes (0.3–9.0 ml). The first signal at 4 nts is the marker that is included in each run. Image [E] shows an overlay of representative electropherograms to illustrate size proportions. The fluorescence unit (FU) represents the signal intensity of small RNAs.

The Bioanalyzer Small RNA Series II Assay (Agilent Technologies, Germany) resolves the small RNA fraction in the size range from 6 to 150 nts. No RNA could be detected in all the samples where Qubit measurement was not possible, irrespective of the initial plasma volume (Figure 1, [A] and [B]). Small peaks in the range of approximately 20 nts could be observed when 6 ml of plasma were extracted with the miRNeasy Serum/Plasma Kit (Figure 1, [C]). Lanes in Figure 1 [D] showed bands in the small RNA area when the presented method with 9 ml of plasma as initial sample input and the miRNeasy Serum/Plasma Kit in combination with the QIAvac system was utilized.

As the applicability of total RNA quantification on the one hand and the analysis of the small RNA fraction on the Bioanalyzer 2100 (Agilent Technologies, Germany) on the other hand was approved after optimization, nine bovine plasma samples were extracted using the isolation procedure as presented. For small RNA-Seq, an input amount of 1–5 µg total RNA is required, due to several quality control checkpoints during library preparation. When working with plasma samples and the aim is the analysis of circulating RNAs, the problem of low abundance of RNAs has to be faced. Due to the limited RNA quantity, library preparation was performed with 35 ng of RNA as starting material as this RNA yield was the least common denominator in RNA concentrations (Table 1).

### Validation of Library Preparation

**Figure 2:**
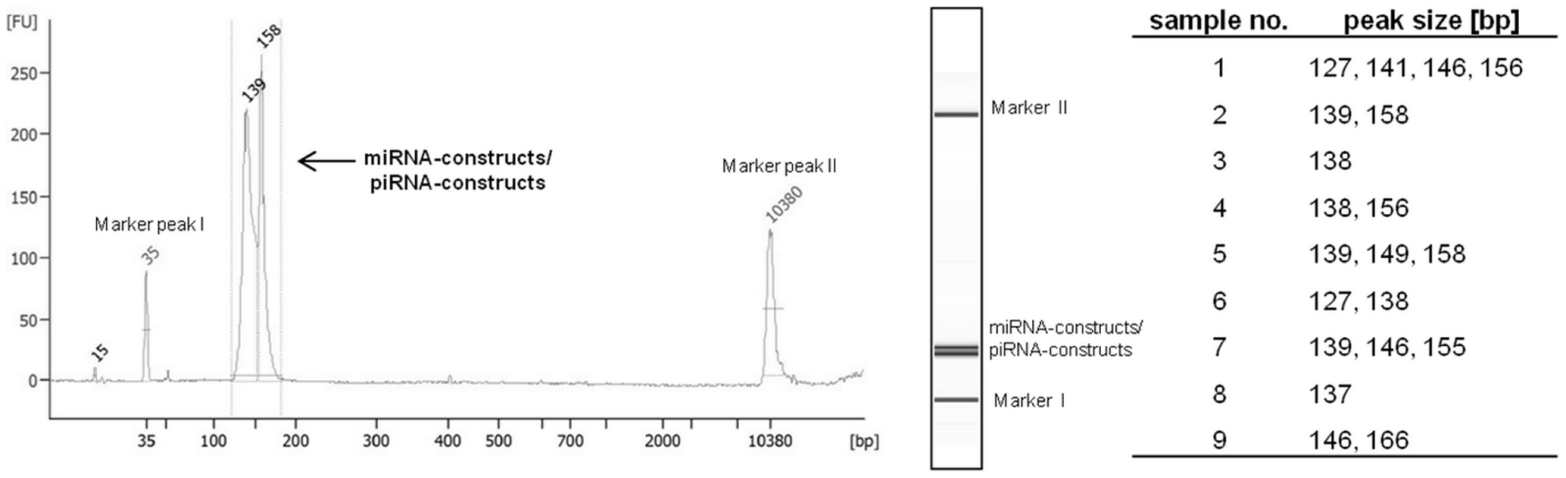
High Sensitivity DNA Bioanalyzer assay as checkpoint for correct size selection during library preparation. All nine samples showed adaptor/RNA/adaptor-constructs in appropriate sizes. One electropherogram is shown as representative example. The lengths of adaptor-ligated constructs from all nine samples were reported as indicated in the column *peak size [bp]*. The initial peak at 35 bp and the final peak at 10.380 bp are marker peaks that are system inherent included in all runs.

During library preparation, adaptors were hybridized to RNAs to bind the resulting sequencing library to oligonucleotides on the flow cell and index sequences were ligated to enable multiplexing of samples. The construct of the 3′-adaptor and the 5′-adaptor with the index sequence has the length of 119 nts. Therefore, depending on the RNA-insert length, a successfully amplified cDNA library depicts miRNA-/adaptor-constructs of approximately 138–144 nts and piRNA-/adaptor-constructs of 144–151 nts. A concluding High Sensitivity DNA Assay on the Bioanalyzer 2100 (Agilent Technologies, Germany) revealed the generation of cDNA libraries with adaptor-ligated constructs in the correct size, which signified the successful amplification of mature miRNAs and piRNAs (Figure 2).

### Sequencing Quality Criteria

For the nine sequenced sample outputs the total phred scores and the average phred scores were calculated. The total phred score is assigned to each nucleotide of the reads and the average phred score is calculated for an entire sequence. For 98.3% of the sequenced nucleotides and for 98.7% of all reads, a Q score >30 was calculated. A Q score of 30 that is assigned to a base implies a probability of an incorrect base call in 1 of 1000 bp [26]. If more than 90% of the reads show an average quality score ≥30, the obtained data is considered to be of high quality regarding the base calling on the HiSeq platform [27]. Additionally, reads were analyzed regarding their proportions of sequences with and without ambiguities. 99.5% of all reads did not contain ambiguities.

### Evaluation of Readcounts

**Figure 3:**
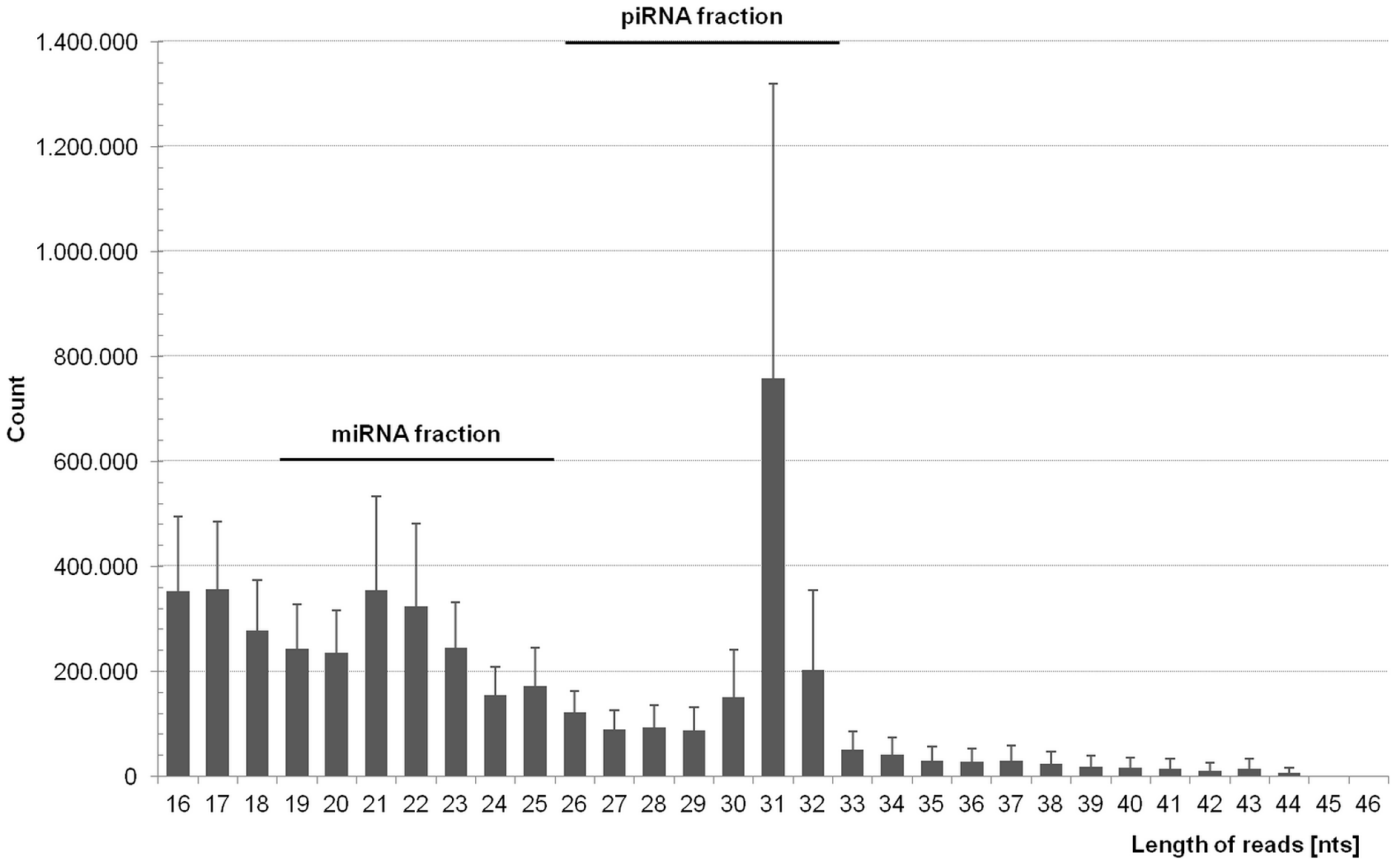
Sequence length distribution pattern analyzed by FastQC software. Calculating the average number of sequences with a certain length for all nine plasma samples, the profile displayed a bimodal pattern. The first peak includes sequences with a length between 20 nts and 24 nts reflecting miRNAs. The second peak indicates the piRNA fraction with sequences of 29 nts to 33 nts in size.

After trimming, data was analyzed in terms of sequence length distribution. The profile in Figure 3 displayed a bimodal pattern with two distinct peaks at 21 nts and 31 nts. The peak in the range of 19 to 25 nts represented the miRNA fraction, while piRNAs were depicted by read lengths between 26 and 32 nts. More readcounts showed a length that is specific for miRNAs (1.728.441±46.787 reads) compared to the number of readcounts that were in the size range of piRNAs (1.505.090±191.338 reads).

**Figure 4:**
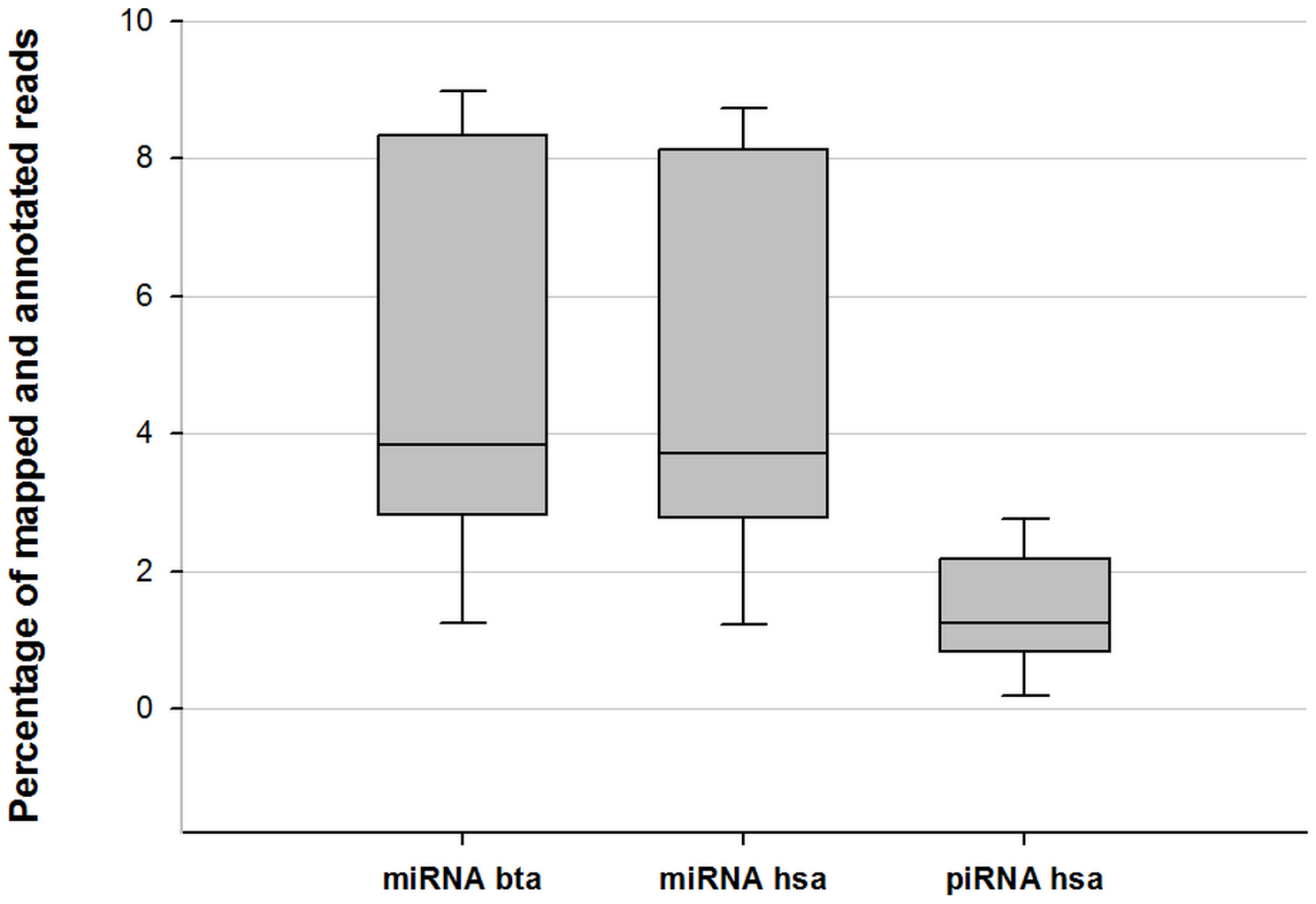
Percentage of mapped and annotated miRNA/piRNA reads compared to the total number of sequences.

**Figure 5:**
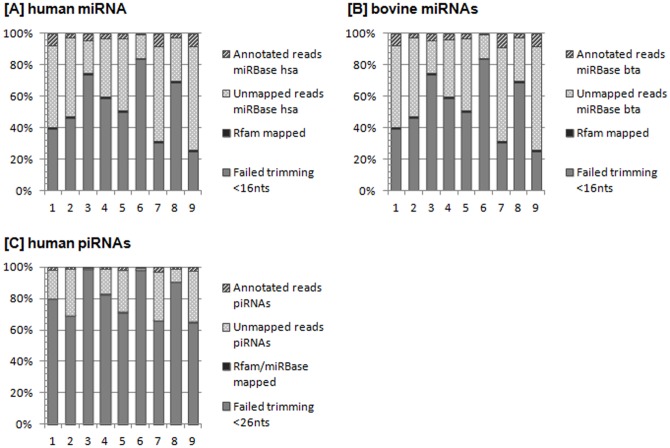
The proportions of trimmed, annotated and non-annotated reads. The total number of sequenced reads (100%) is divided into reads that failed trimming and reads that passed trimming and were mapped to Rfam database. Reads that were not mapped to Rfam database, were mapped to miRBase. Reads separated into annotated reads in miRBase and in reads that failed miRNA annotation. Image [A] displays miRNA results from mapping to human reference indexes. Image [B] presents miRNA results from mapping to bovine references. Regarding piRNAs (Image [C]), reads that could not be mapped to miRBase were aligned to piRNA database. They separate into annotated piRNAs and unmapped piRNAs.

The sequenced samples contained on average 10.465.348±3.638.968 reads (Table 2). 4.508.918±1.490.660 sequences passed the trimming quality threshold of 16 nts length, while 5.956.429±3.874.215 sequences failed the trimming process and were not further included in the data analysis steps (Table 2). 114.566±43.523 sequences were mapped to Rfam database, removing RNAs that were neither miRNAs nor piRNAs, but rRNAs, snRNAs, snoRNAs and tRNAs. Contrarily, sequences that were not mapped to Rfam database (4.394.352±1.467.749 reads) were processed in the search for annotated miRNAs using miRBase. miRBase sequence database provides 2.578 entries representing human mature miRNAs and 783 entries for bovine mature miRNAs. Mapping data to the hsa entries in miRBase, 471.640±269.100 reads were annotated, being a proportion of 4.8±2.8% of the total sequenced reads (Figure 4). Setting the threshold of sequenced readcounts to 50 reads at an average over all animals, 99.2% of annotated human miRNAs were abundant with more than the defined threshold. 482.072±274.637 reads could be mapped to annotated bta miRNA sequences accounting for a 5.0±2.9% share of the total readcounts (Figure 4). 99.5% of annotated reads showed more than 50 readcounts at an average over all sequenced samples. 3.467.882±1.212.523 reads remained unmapped to no database. A detailed summary of evaluated miRNA reads is given in Table 2 and Figure 5 [A] and [B] clarifies the proportions of trimmed, annotated and remaining readcounts.

**Table 2:** miRNA data analysis shows the composition of evaluated reads from nine animals generated by computer data analysis pipeline using free software tools.

Data evaluation - pipeline miRNA	Animal 1	Animal 2	Animal 3	Animal 4	Animal 5	Animal 6	Animal 7	Animal 8	Animal 9	MEAN	SD
Total sequences	9.694.743	9.585.690	18.089.452	11.225.200	9.210.634	13.046.396	9.871.768	8.900.340	4.563.907	10.465.348	3.638.968
Passed trimming >16 nts	5.942.478	5.189.172	4.797.229	4.656.921	4.655.609	2.164.606	6.894.507	2.826.235	3.453.508	4.508.918	1.490.660
Failed trimming <16 nts	3.752.265	4.396.518	13.292.223	6.568.279	4.555.025	10.881.790	2.977.261	6.074.105	1.110.399	5.956.429	3.874.215
Rfam mapped	155.108	95.216	207.425	102.996	96.956	78.487	132.335	92.173	70.402	114.566	43.523
Rfam unmapped	5.787.370	5.093.956	4.589.804	4.553.925	4.558.653	2.086.119	6.762.172	2.734.062	3.383.106	4.394.352	1.467.749
Annotated reads miRBase bta	764.079	283.405	840.542	430.832	334.847	162.123	885.241	237.019	400.560	482.072	274.637
% (/total sequences)	7.9	3.0	4.6	3.8	3.6	1.2	9.0	2.7	8.8	5.0	2.9
Unmapped reads miRBase bta	5.023.291	4.810.551	3.749.262	4.123.093	4.223.806	1.923.996	5.876.931	2.497.043	2.982.546	3.912.280	1.269.337
Annotated reads miRBase hsa	752.137	281.446	827.746	416.990	323.844	159.927	861.539	233.927	387.208	471.640	269.100
% (/total sequences)	7.8	2.9	4.6	3.7	3.5	1.2	8.7	2.6	8.5	4.8	2.8
Unmapped reads miRBase hsa	5.035.233	4.812.510	3.762.058	4.136.935	4.234.809	1.926.192	5.900.633	2.500.135	2.995.898	3.922.711	1.273.750

Trimming data sets with the length threshold of 26 nts resulted in 1.792.803±1.125.965 reads that passed and 8.672.545±4.295.024 reads that failed (Table 3). Sequences that were not mapped to Rfam and miRBase were mapped to 32.046 entries in NCBI's piRNA database, resulting in 128.333±69.954 matching human piRNA reads. Compared to the total number of sequenced reads, 1.4±0.8% of reads were assigned to be piRNAs (Figure 4). 98.5% of annotated piRNAs showed at an average over all animals more than 50 readcounts. Due to the fact that no bovine piRNAs are reported yet, there is no matching annotation reference library available. A detailed summary of evaluated piRNA reads is given in Table 3 and Figure 5 [C] clarifies the proportions of trimmed, annotated and remaining readcounts. MiRNA and piRNA readcounts normalized to library sizes are presented in Table S1.

**Table 3:** piRNA data analysis shows the composition of evaluated reads from nine animals generated by computer data analysis pipeline using free software tools.

Data evaluation - pipeline piRNA	Animal 1	Animal 2	Animal 3	Animal 4	Animal 5	Animal 6	Animal 7	Animal 8	Animal 9	MEAN	SD
Total sequences	9.694.743	9.585.690	18.089.452	11.225.200	9.210.634	13.046.396	9.871.768	8.900.340	4.563.907	10.465.348	3.638.968
Passed trimming>26 nts	1.992.987	3.002.846	313.300	1.977.291	2.675.451	303.727	3.385.648	871.336	1.612.639	1.792.803	1.125.965
Failed trimming <26 nts	7.701.756	6.582.844	17.776.152	9.247.909	6.535.183	12.742.669	6.486.120	8.029.004	2.951.268	8.672.545	4.295.024
Rfam/miRBase mapped	24.229	27.254	10.254	15.375	23.495	4.166	34.196	9.794	14.273	18.115	9.723
Rfam/miRBase unmapped	1.968.758	2.975.592	303.046	1.961.916	2.651.956	299.561	3.351.452	861.542	1.598.366	1.774.688	1.116.766
Annotated reads piRNAs	144.667	89.786	33.649	140.601	190.090	101.363	271.987	77.665	105.186	128.333	69.954
% (/total sequences)	1.5	0.9	0.2	1.3	2.1	0.8	2.8	0.9	2.3	1.4	0.8
Unmapped reads piRNAs	1.824.091	2.885.806	269.397	1.821.315	2.461.866	198.198	3.079.465	783.877	1.493.180	1.646.355	1.065.235

**Figure 6:**
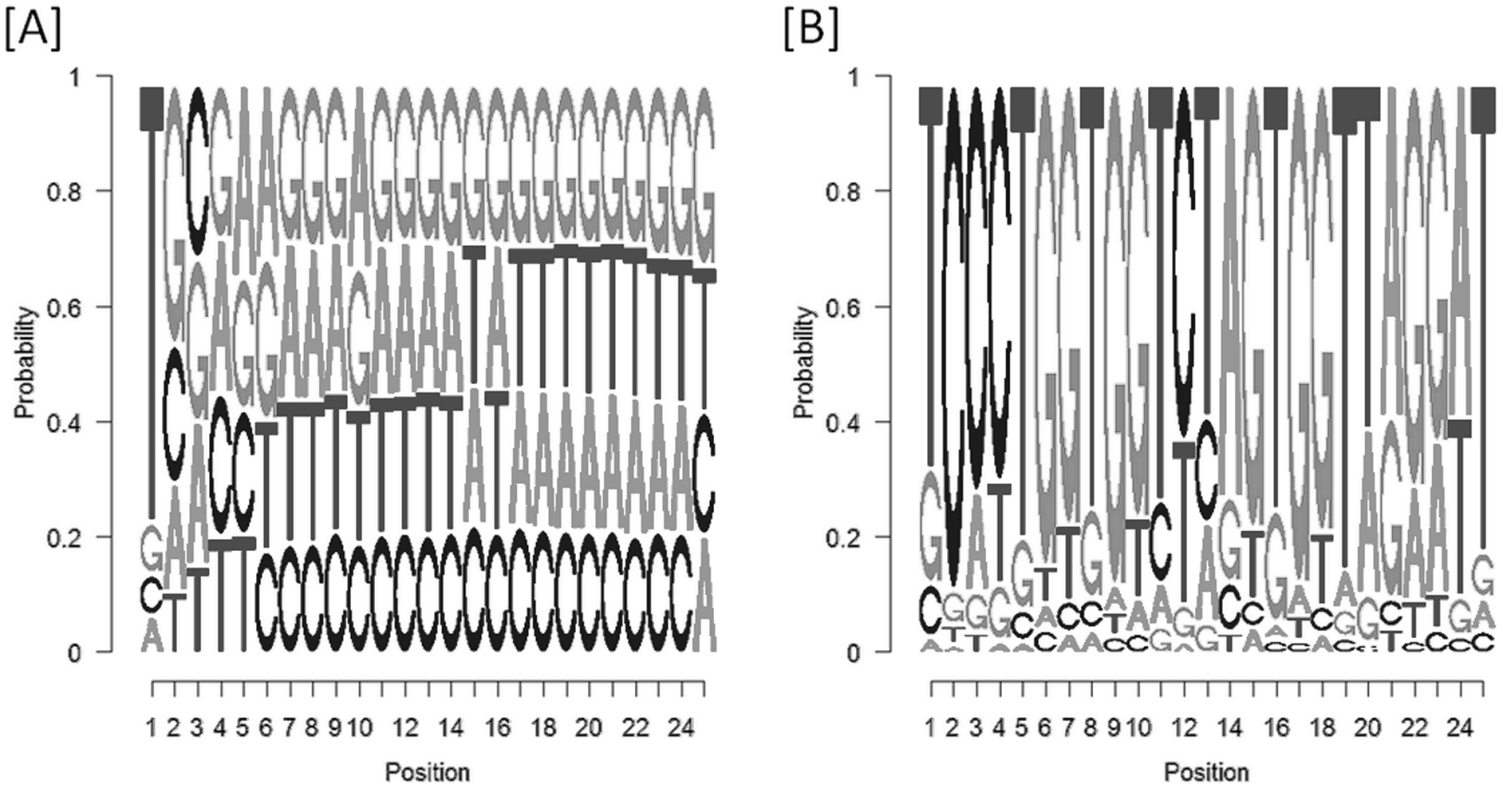
Sequence motif analysis to evaluate piRNA 5′-T-bias. Image [A] shows the piRNA reference that was used for alignment and Image [B] represents the nucleotide composition of mapped piRNAs pointing out the T-bias at 5′.

According to the piRNA biogenesis model, the ping-pong model, piRNAs can be divided into primary and secondary piRNAs [28]. Primary piRNAs have a strong tendency for 5′ bias for uridine and do not have nucleotide bias at position 10, while secondary piRNAs have a bias for adenine (A) at position 10 but do not show a 5′ bias. Results of the sequence motif analysis are displayed in Figure 6. 69.1% of analyzed piRNA readcounts showed a thymin (T) at the first nucleotide and 5.2% an A at position 10. The human piRNA reference that was used for piRNA alignment also showed 5′-T bias (79.0%) that mirrors expediency of this dataset. However, reference piRNAs were not weighted compared to the sequenced samples. The sequence motif with the highest probability is arising from the top 4 piRNAs that account for 66.2% of mapped piRNAs (Table S2). Computing sense and antisense piRNA overlaps, no ping-pong signatures could be identified. Using proTRAC software, no clusters could be identified in the bovine genome.

## Discussion

### Extraction Procedure

MiRNAs have shown to be powerful candidates in biomarker profiling for the detection of diseases or altered health conditions. Circulating RNAs hold great promise in finding new biomarkers not only in tissues but also in other body matrices like the easy-to-sample plasma. Analyzing circulating RNAs compared to cell-bound RNAs is a new approach in the screening for biomarkers. For the analysis of the transcriptome in plasma, a suitable isolation method is needed to perform reliable and holistic small RNA-Seq experiments. Several prevalent RNA isolation systems on the market were given a trial for the extraction of circulating RNAs from plasma samples. The initially tested NucleoSpin miRNA Plasma Kit from Macherey-Nagel as well as the Qiagen miRNeasy Mini Kit with a supplementary protocol could not provide measurable RNA yields. Extracting the starting volume of 9 ml with the NucleoSpin miRNA Plasma Kit was not performed, because the columns were not compatible with the vacuum device. However, plasma input was raised up to a fourfold than recommended, but higher volumes led to column clogging. Furthermore, during optimization process, Qiagen launched an adapted isolation kit for RNAs especially from plasma or serum matrices. Since the extraction method with this miRNeasy Serum/Plasma Kit using 9 ml of plasma provided sufficient RNA yields and good quality NGS data, it was omitted to test the obsolete miRNeasy Mini Kit system with the supplementary protocol.

Yields of the extracted RNAs fluctuated between the analyzed samples (Table 1). It has to be considered, that RNA was extracted from nine different bull calves and total RNA content may vary between animals due to a different health status, immune situation, genetic variation or other stimuli.

Analyzing the small RNA fraction on the Bioanalyzer 2100 (Agilent Technologies, Germany), double-peaks or shifted peaks towards a higher nucleotide length, as it can be seen in Figure 1 [D], indicated the presence of not solely miRNAs but also of slightly longer RNAs. Library validation reinforced this assumption (Figure 2) and mapping finally confirmed the presence of miRNAs as well as piRNAs (Table 2, Table 3).

### cDNA Library Preparation

The produced cDNA libraries were tested for the right size and purity using a High Sensitivity DNA chip on the Bioanalyzer 2100 (Agilent Technologies, Germany). The adaptor-ligated constructs varied in size, depending on the length of the initial RNA fragment. Inserts derived from miRNAs with a length of approximately 19–25 nts result in constructs of 138–143 bp. For piRNAs, the corresponding fragment length would be 144–151 bp, as the insert RNA has 25–32 nts. Although there was not a marked peak in the size of >138 bp in all of the samples (Figure 2), peaks had a broad basis covering the range not only of miRNAs but also of slightly longer small RNAs. However, despite of the correct size of amplified constructs, no statement about the variety of the containing RNAs in a sample could be made at that point.

### Sequencing and Mapping

To screen the abundance of piRNAs and mature miRNAs in bovine plasma, sequencing data were mapped to miRBase and NCBI's piRNA database according to the presented data analysis strategies. This revealed the presence of 4.8±2.8% of annotated human miRNAs, 5.0±2.9% of annotated bovine miRNAs and 1.4±0.8% of annotated human piRNAs in plasma samples (Figure 4, Table 2 and Table 3). It is known that piRNAs act mainly in Piwi-dependent transposon silencing, heterochromatin modification and in germ cell maintenance [29]. They were discovered in rat testes [30], but their presence was also confirmed in somatic tissues of fruit fly, mouse and *rhesus macaque* [31]. Thus, since their discovery in 2006, verification of their presence in various tissues and their role in different, not only germ-line affecting functions is gaining significance. It was recently shown that piRNAs were also found in the circulation, namely in exosomes, endosome-derived membrane microvesicles that contain specific RNA transcripts and are thought to be involved in cell-cell communication. Compared to results in human exosomes, which contained 1.31% of piRNAs, our findings in bovines are in the same dimension (Table 3) [32]. Exosomal miRNAs were represented in humans as the major part (76.2% of mappable miRNAs) [32], while circulating miRNAs in bovine plasma showed a presence of approximately 5% (Figure 4). Noticing that these are results from different species, the question why the miRNA content differs between exosomes and pure plasma gives rise to several assumptions. Plasma as analysis matrix is very sensitive to RNase digestion. MiRNAs are not only present in secure exosomes but also free in circulation and/or bound to proteins. As many reads did not pass the trimming due to a too short length (<16 nts), it is conceivable that the RNA degradation was already in an advanced stage (Figure 5, Table 2). However, the analysis of circulating miRNAs or piRNAs is still potential, due to a substantial number of mapped readcounts for each miRNAs and piRNAs (Table 2, Table 3). Hence, it still needs to be elucidated, if there is a balanced level of circulating small RNAs and if the miRNA/piRNA concentration in plasma is actively regulated by organisms. Moreover, clarification is needed to what extend miRNAs/piRNAs are present as apoptotic by-products or through active release.

Williams et al. reported a top 10 list of the most abundant circulating miRNAs in plasma samples from human volunteers using small RNA sequencing [33]. Comparing these results with our data collected by small RNA-Seq of bovine plasma samples, five out of ten miRNAs (miR-486, miR-21, miR-22, miR-25 and miR-92) were as well present in our top 20 list of most abundant miRNAs, either for the analyzed miRNA profile through mapping to hsa and bta miRBase entries (Table S3). Running the BLASTN algorithm (http://blast.ncbi.nlm.nih.gov, version 2.2.29, human genomic plus transcript database) revealed high query coverages (bta-miR-486: 46%, bta-miR-21: 100%, bta-miR-22 100%, bta-miR-25 100%, bta-miR-92: 95%) and therefore high similarity of bta-miRNAs aligned to human transcripts. The human most abundant miRNA that was reported by Williams et al. [33] is miR-451 (∼50%), a red blood cell specific miRNA which is present in our dataset to 0.5% (Table S1 [A]). The second (miR-486) and third (miR-92a) most abundant human miRNAs are found in the top 10 list of sequenced bovine miRNAs (Table S3). The major miRNA in bovine plasma (miR-122) was not ranked amongst the top 10 human miRNAs. MiR-423-3p and miR-423-5p were ranked number 3 and 4 and were as well not present in the human top 10. Apart from pathological processes like hepatic cell death [34], heart failure [35] and type-2 diabetes [36], no relations to physiological conditions could be found in literature that could explain altered miRNA compositions between humans and bovines. It remains unclear to what extend findings in humans can be transferred to bovines and vice versa due to great differences in their physiologies, e.g. the digestive system.

Clearly, there is an inconsistency in the number of mapped and annotated readcounts compared to reads that have the appropriate length of miRNAs and piRNAs, respectively (Figure 3, Table 2 and Table 3). Furthermore, there is a considerable part of a) sequenced reads that failed the trimming criterions and b) of small RNAs that could not be mapped at all (Figure 5). First, possibly there are more small RNAs in the samples that are not annotated yet. Second, as mentioned earlier, RNAs are exposed to a high content of RNases in plasma. Even though miRNAs are known to be relatively stable in plasma [4], other circulating RNAs, e.g. from apoptotic cells or active release, could be degraded to pieces with an equivalent size range and superimpose the length distribution profile.

Although no clusters could be found along the genome and neither a bias for an A at position 10 nor ping-pong structures could be observed, the indication for primary piRNAs remain considerable. Besides appropriate length distribution in combination with mapping to piRNA reference datasets, the piRNA sequence motif analysis raised evidence that bovine plasma contained primary piRNAs with a characteristic T nucleotide enrichment at 5′ (Figure 6 [B]). In addition, a 10 nt A bias was primarily observed in *drosophila melanogaster*, but not in mammals like *rhesus macaque* or *mus musculus* [31]. This signifies the knowledge gap in the role of piRNAs and their specific features in the mammalian circulation.

## Conclusions

In conclusion, the amount of total RNA that is extracted using commercially available isolation kits for circulating RNAs from body fluids is only sufficient for RT-qPCR measurements but not for NGS analyses. Therefore, the presented optimized extraction procedure for plasma samples was developed to enable Next Generation small RNA Sequencing. The miRNeasy Serum/Plasma Kit (Qiagen, Germany) was used in combination with the QIAvac vacuum device to process the increased sample volumes (starting material  = 9 ml plasma). Plasma is low RNA concentration material. This has to be kept in mind during the experimental design of animal trials to ensure a sufficient available sample volume.

NGS is a very fast developing and yet a highly advanced technology. Companies offer a multitude of isolation kits for diverse applications (e.g. specialized kits for different sample origins, RNA types that are to be analyzed, starting sample amount, pre-analysis storage of samples, etc.) and library preparation kits provide increasingly improving protocols for faster procedures with shorter hands-on times in the lab and less required starting material. Sample preparation and bioinformatics in the downstream data analysis are yet mostly not designed and certified for other samples than human. Hence, there is still a need for more specific requirements. However, the presented method enables the small RNA-Seq analysis of circulating, cell-free miRNAs and piRNAs in bovine plasma with good performance data and a substantial number of further usable readcounts, e.g. for differential gene expression profiling, although the proportion of miRNAs together with piRNAs on total readcounts is not higher than 10% (Figure 4, Table 2 and Table 3).

As mentioned above, plasma as minimally invasive sample could make the biomarker profiling highly attractive for example in molecular diagnostics, risk assessment or food safety [37]. This experiment exhibited new insights in the composition of bovine circulating small RNAs and described the presence of piRNAs. Consequently, better knowledge about piRNAs and their analysis could potentially lead to find biomarkers on other RNA levels than mRNAs or miRNAs. Besides plasma, other body fluids like urine, milk or saliva could also be suitable non-invasive biomarker matrices. Investigating the miRNA and/or the piRNA profile in bio-fluids via small RNA-Seq could be a new option to detect novel biomarker signatures.

## Supporting Information

Table S1 **[A] bovine miRNA readcounts, [B] human miRNA readcounts and [C] human piRNA readcounts normalized to library sizes in reads per million (rpm).**
(XLSX)

Table S2 **Top 15 piRNAs mainly contributing to the sequence motif analysis in** **Figure 6** **[B].**
(XLSX)

Table S3 **Top 20 ranking list displays the most abundant circulating miRNAs in nine bovine plasma samples using small RNA-Seq.** Data was compared to findings by Williams et al. [33]. Same miRNAs are marked in bold type. Table S3 [A] reports data from mapping to bovine entries in miRBase, Table S3 [B] lists results for mapping to human reference index.
(XLSX)
